# Supplementary material for: Simultaneous enhancement of multiple functional properties using evolution-informed protein design
Source: Nat Commun. 2024 Jun 20;15:5141. doi: 10.1038/s41467-024-49119-x (PMC11190266; doi:10.1038/s41467-024-49119-x)
Supplement: Supplementary file 1 — Supplementary Information [file 41467_2024_49119_MOESM1_ESM.pdf]

Supplementary Information

Simultaneous Enhancement of Multiple Functional Properties Using Evolution-informed Protein Design

Benjamin Fram[1,2,✉], Yang Su[1,*], Ian Truebridge[3,4,5,*], Adam J. Riesselman[1,6], John B. Ingraham[1], Alessandro Passera[2,7], Eve Napier[8], Nicole N. Thadani[1,9], Samuel Lim[1], Kristen Roberts[10], Gurleen Kaur[10], Michael A. Stiffler[2,11], Debora S. Marks[1,12], Christopher D. Bahl[3,4,5], Amir R. Khan[8,13], Chris Sander[1,2,12], and Nicholas P. Gauthier[1,2,12,✉]

[1]Department of Systems Biology, Harvard Medical School, Boston, MA, USA
[2]Department of Data Sciences, Dana-Farber Cancer Institute, Boston, MA, USA
[3]Institute for Protein Innovation, Boston, MA, USA
[4]Division of Hematology/Oncology, Boston Children's Hospital, Harvard Medical School; Boston, MA, USA
[5]current address: AI Proteins; Boston, MA, USA
[6]Program in Biomedical Informatics, Harvard Medical School, Boston, MA, USA
[7]current address: Research Institute of Molecular Pathology (IMP), Vienna BioCenter (VBC), Campus-Vienna-Biocenter 1, 1030 Vienna, Austria
[8]School of Biochemistry and Immunology, Trinity College Dublin, Dublin 2, Ireland
[9]current address: Apriori Bio, Cambridge, MA, USA
[10]Selux Diagnostics, Inc., 56 Roland Street, Charlestown, MA, USA
[11]current address: Dyno Therapeutics, 343 Arsenal Street, Watertown, MA, USA
[12]Broad Institute of MIT and Harvard, Cambridge, MA, USA
[13]Division of Newborn Medicine, Boston Children's Hospital, Boston, MA, USA
*Y.S. and I.T. share joint second authorship
✉Correspondence should be addressed to B.F. (benjamin.fram.research@gmail.com) and N.P.G. (nicholas.gauthier.research@gmail.com)

List of Figures

S1 Correlation of predicted fitness (EVH) with experimentally-determined fitness effect 3
S2 Design predicted fitness (EVH) versus randomly generated sequences 4
S3 Multiple sequence alignment of each design with their most similar homologs 5
S4 Properties of mutated versus non-mutated positions 6
S5 Conservation and surface accessibility at each position relative to mutation count 7
S6 Design mutations mapped to structure of WT TEM-1 8
S7 Additional ampicillin resistance assays 9
S8 Nitrocefin hydrolysis - product concentration over time 10
S9 Nitrocefin hydrolysis - Michaelis-Menten fit to measured data 11
S10 Ampicillin hydrolysis - substrate concentration over time 12
S11 Differential Scanning Fluorimetry (DSF) melt curves 13
S12 MIC determination of β-lactams for which the designs were similar to negative controls . 14
S13 Additional aztreonam, ceftazidime, and cephalothin resistance assays 15
S14 Composite omit maps of refined X-ray structures 16
S15 Difference Distance Matrix of 80.a versus WT TEM-1 17
S16 Difference Distance Matrix of 80.b versus WT TEM-1 18
S17 Difference Distance Matrix of 70.a versus WT TEM-1 19
S18 Structural assessment of active site in X-ray crystal structures to WT TEM-1 20
S19 Experimentally-determined fitness effect of mutations in WT TEM-1 21
S20 The predicted fitness of G251W with other 70.a mutations 22
S21 Focused assessment of double mutations with G251W 23
S22 Structural assessment of the G251W mutation in 70.a 24

List of Tables

S1 Nitrocefin Kinetics - Michaelis-Menton parameters 25
S2 β-Lactamase crystallographic data and refinement statistics 26

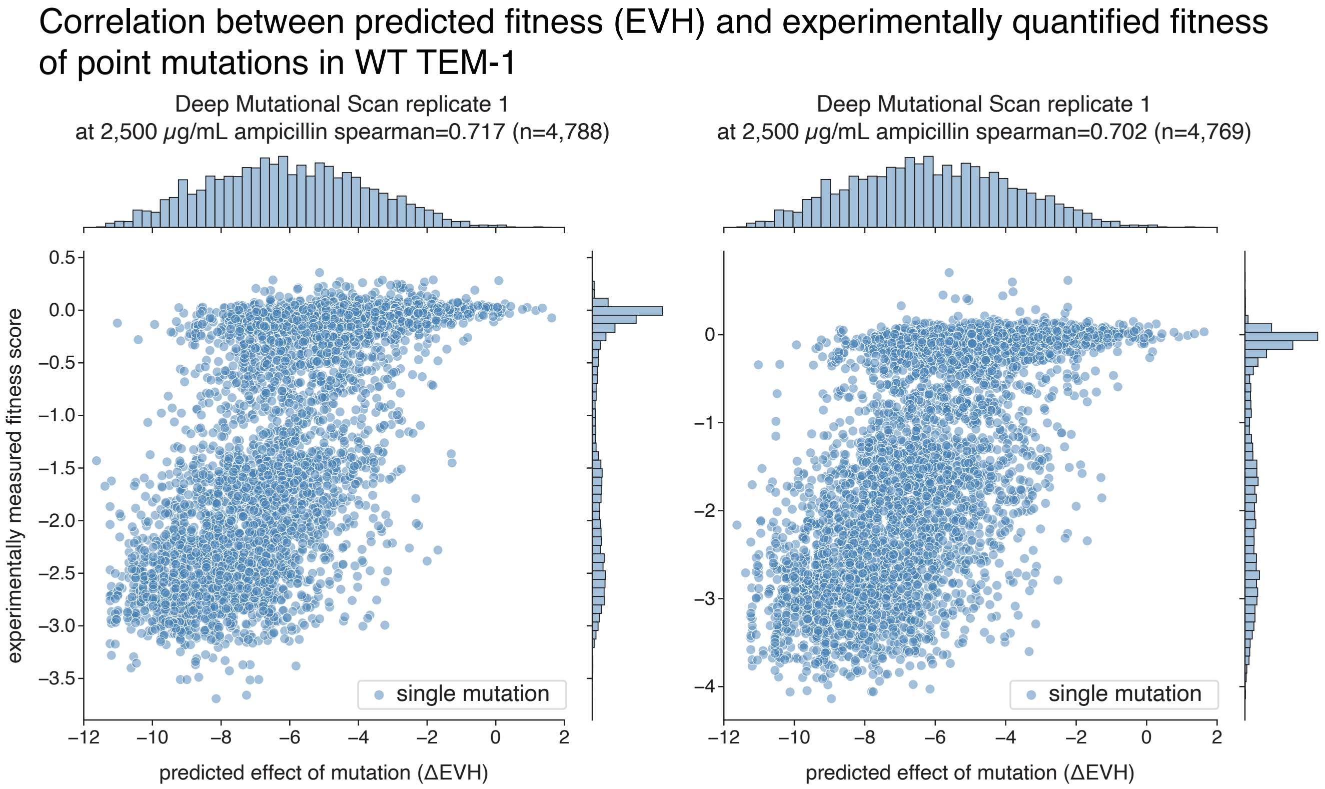

Supplementary Figure 1. Relationship between predicted fitness score and experimentally-determined fitness effect of individual mutations from a published deep mutational scan[1]. Each blue dot represents a single point mutation in WT TEM-1 of which there are 4,788 possible mutations over 252 positions aligned in the multiple sequence alignment used for model generation and fitness prediction. The predicted fitness effect (ΔEVH) on the x-axis is the predicted fitness of the point mutant minus the predicted fitness of WT TEM-1. The experimentally measured fitness score on the y-axis is defined in Stiffler et al.[1]. **Left**: Experimental replicate 1, which quantified all possible mutations (n=4,788 with spearman=0.717). **Right**: Experimental replicate 2, which quantified all mutations in 251 positions (i.e, one fewer than replicate 1; n=4,769 with spearman=0.702). Source data are provided in the Source Data file.

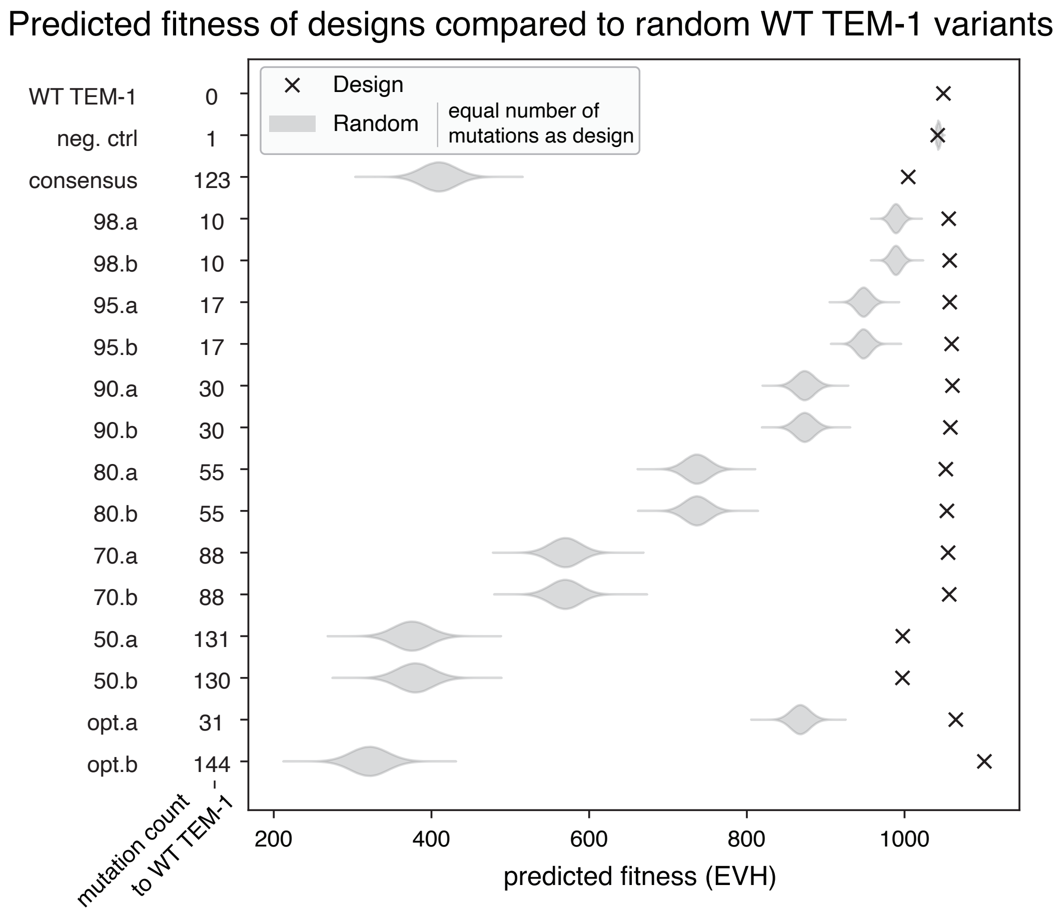

Supplementary Figure 2. Comparison of each designs' predicted fitness with randomly generated sequences. For each design, 1 million random protein sequences were generated on the WT TEM-1 sequence background with an equal number of mutations as the design. The predicted fitness of each design is indicated as an "X" and the distribution of the random sequences is shown as a violin plot. In general, the designs had a much higher predicted fitness than random variants. Example source data are provided in the Source Data file. Random data can be regenerated using scripts at https://github.com/gauthierscience/beta-lac-protein-design[2].

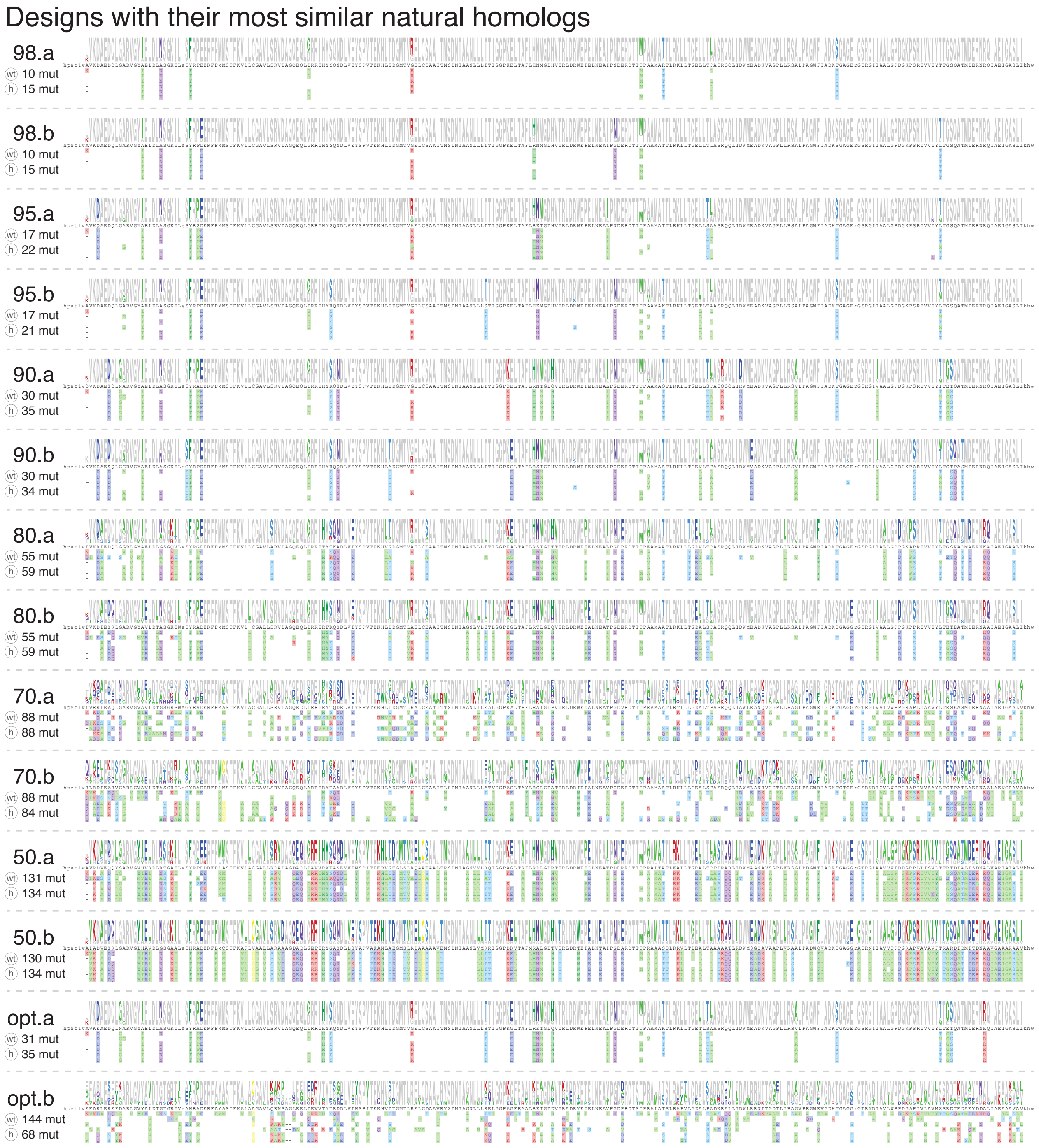

Supplementary Figure 3. Multiple sequence alignments (MSAs) of each tested design with WT TEM-1 and their most similar homologs in the MSA used for model inference. For each design, homologs from the MSA were ranked by sequence identity (number of identical amino acids at each position), and the top five were selected. Each row contains a single design with the design sequence followed by WT TEM-1 and the ordered list of most similar homologs (most similar at the top). Mutations counts of the design relative to WT TEM-1 (wt) and the most similar homolog (h) are listed under the design name. Amino acids changes relative to the design are colored by new residue property (standard colors: green, hydrophobic and glycine (G); blue, negative charge; red, positive charge; light blue, polar). The logo for each position follows the same color scheme, with the design amino acid shown in gray. Dashs (-) represent gaps. Alignments generated by https://fast.alignmentviewer.org. Source data are provided in the Source Data file.

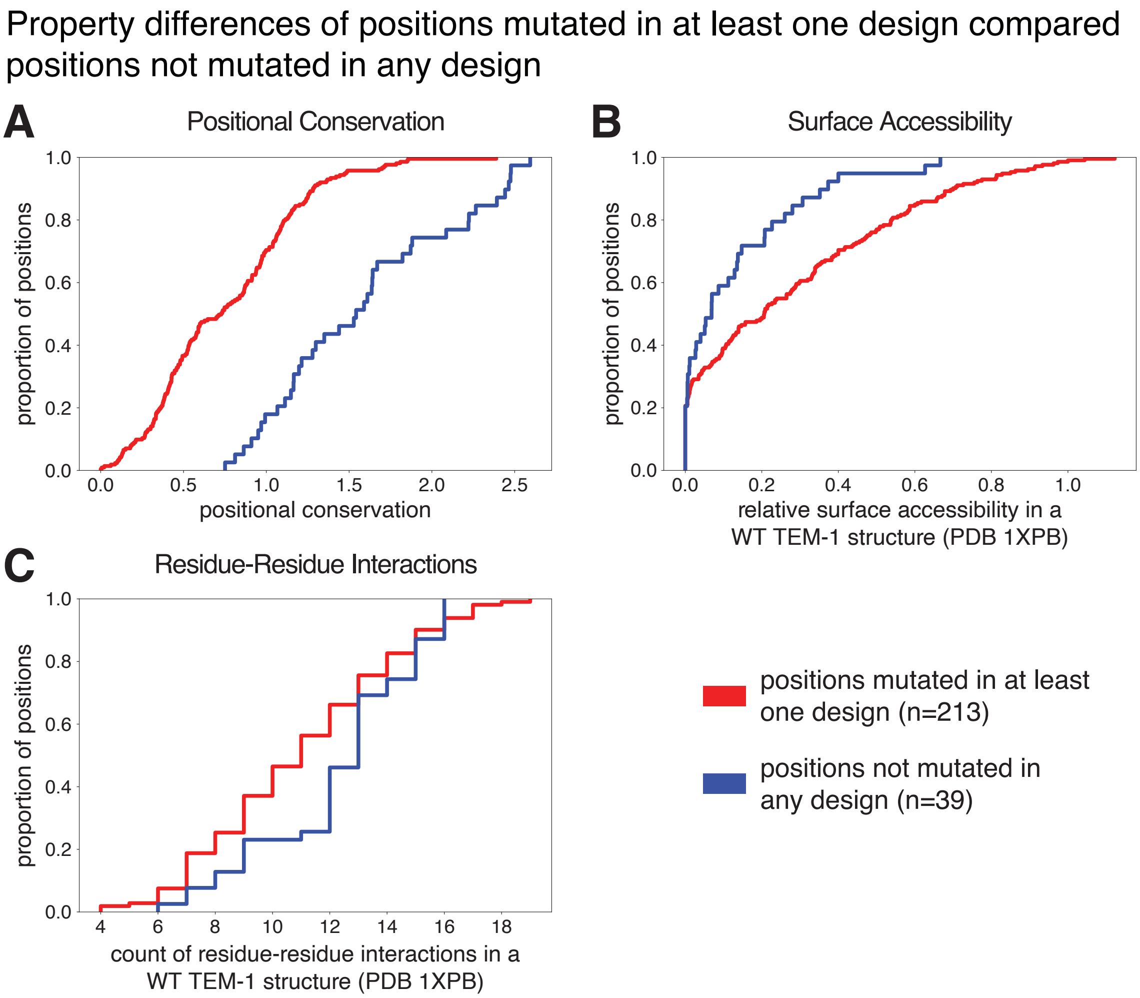

Supplementary Figure 4. Properties of positions mutated in any of the generated designs compared to non-mutated positions. Each panel contains two cumulative distribution functions (CDFs): positions mutated in one or more designs (red) and positions not mutated in any design (blue). Mutated and non-mutated positions were aggregated from all 38 of the generated designs: opt.a, opt.b, and the six designs generated for each of the six distance constraints (i.e., not only the designs that were experimentally characterized). The y-axis is the proportion of positions that are equal or less than the value of the property shown on the x-axis. **[A]** CDFs of positional conservation in the multiple sequence alignment used for model inference (maximum shannon entropy of all positions minus the shannon entropy at each position). Positions mutated in the designs were less likely to be conserved than positions not mutated. **[B]** CDFs of relative surface accessibility from DSSP analysis (*ACC* field) of a published WT TEM-1 structure (PDB: 1XPB). Residues mutated in the designs were more likely to be surface accessible than non-mutated residues. **[C]** CDFs of residue-residue interaction counts from a published WT TEM-1 structure (PDB: 1XPB). An interaction is defined as having any atom in one residue be within 5 angstroms of any atom in the other residue. Residues mutated in the designs generally had fewer interactions than non-mutated residues. Source data are provided in the Source Data file.

Properties of mutated positions: comparison of mutation count, positional conservation, and surface accessibility for each set of distance-constrained designs

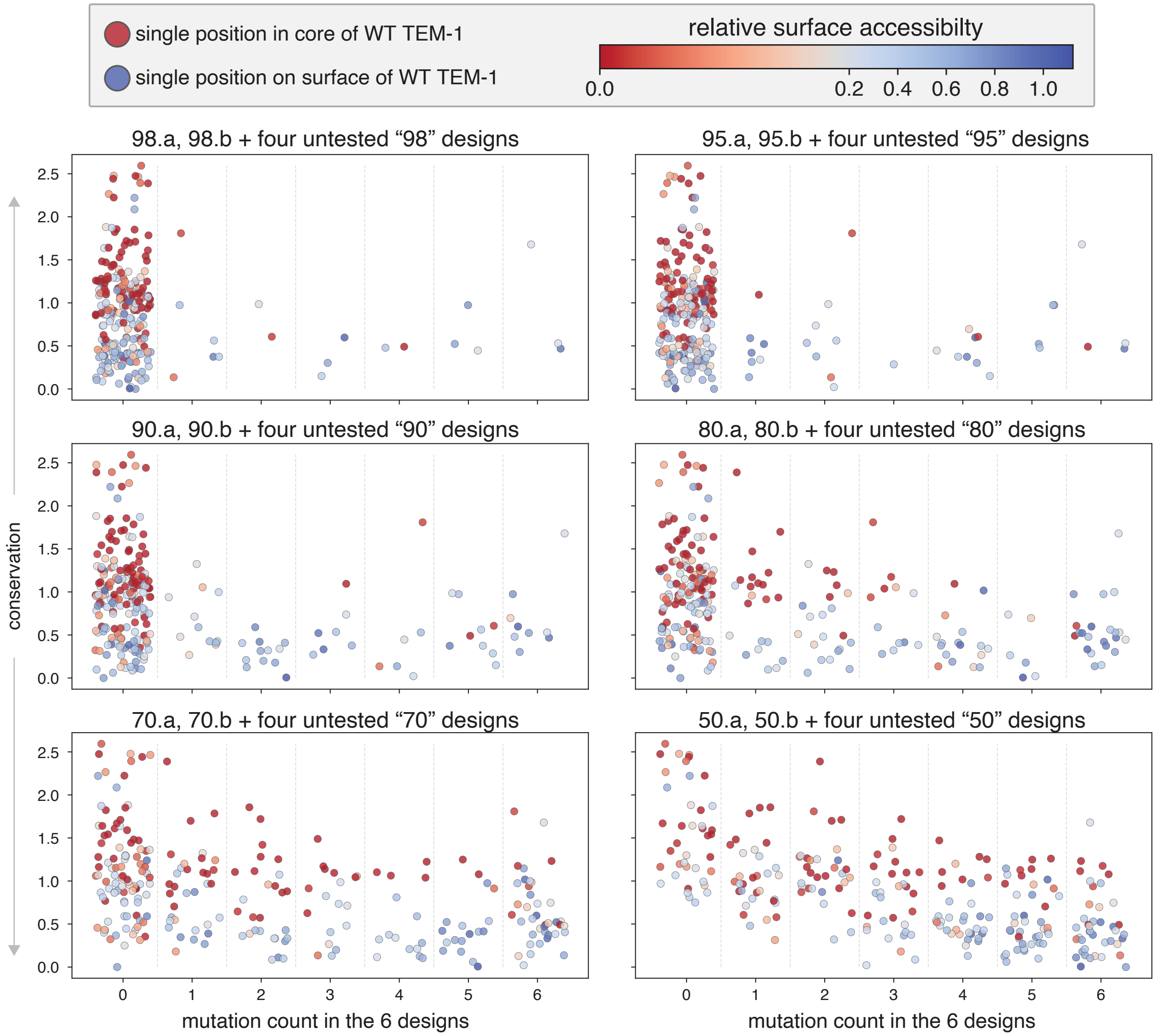

Supplementary Figure 5. Relationship of mutation count at each position to conservation and surface accessibility. Each dot represents a single position that was aligned in the natural multiple sequence alignment and available to be mutated in the design process (n=252). Each plot contains data from all six design sequences at the specified distance threshold (i.e., 98, 95, 90, 80, 70, 50). [X-axis]:the mutation count for each position tallied across the six designs. [Y-axis]: conservation as defined as the maximum shannon entropy of all positions minus the shannon entropy at each position. [Colors]: each dot (position) is colored by the relative surface accessibility of the position in the WT TEM-1 structure (PDB: 1XPB). Red indicates fully buried in the core of the protein and dark blue represents maximum surface accessibility. Intermediate accessibility is colored as shown in the colorbar, with the transition between red and blue set to the median surface accessibility value so that half of the positions are core and half exposed (i.e., half red and half blue). Source data are provided in the Source Data file.

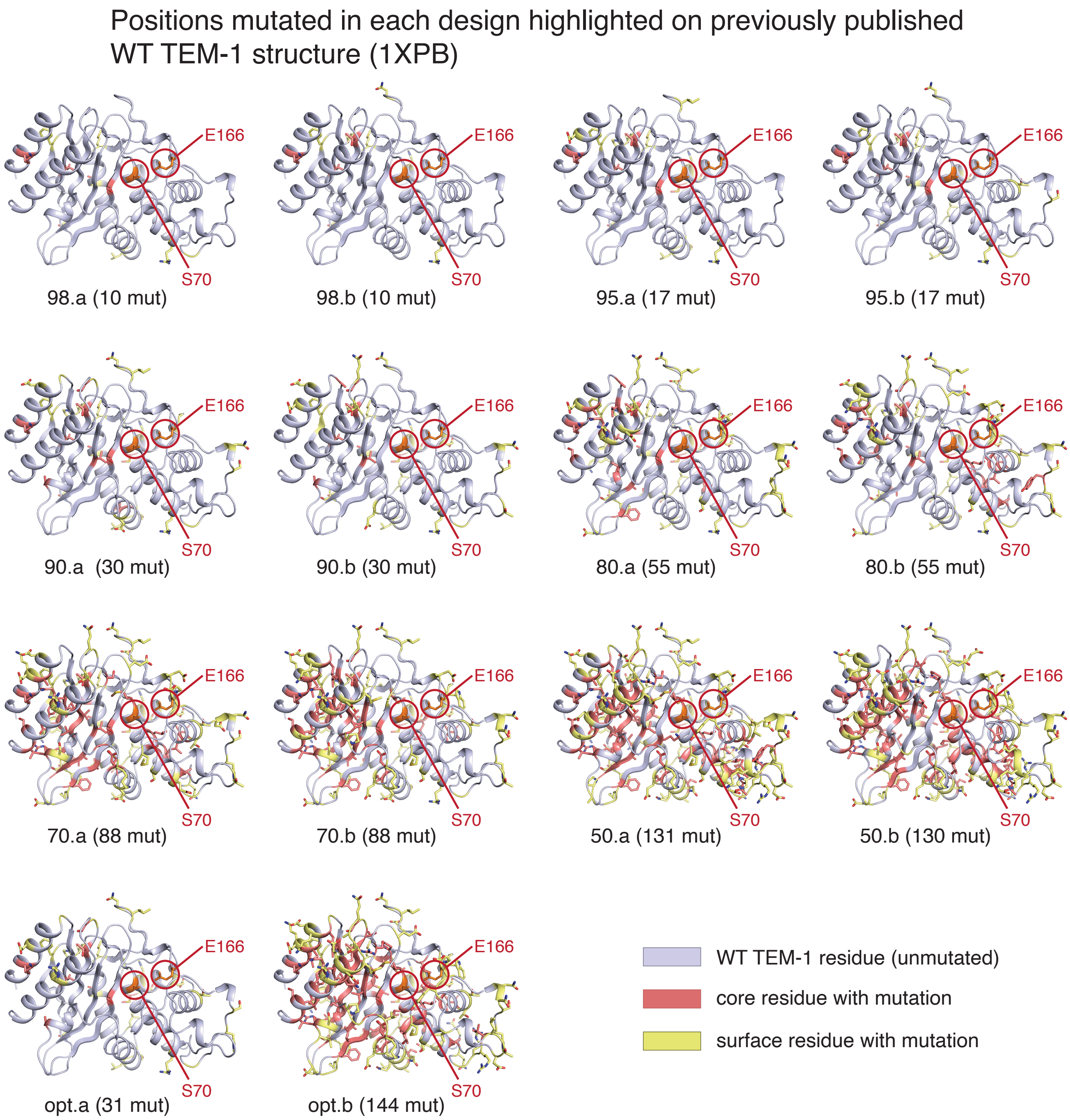

Supplementary Figure 6. Structural representation showing the positions mutated in each design on a published WT TEM-1 structure (PDB: 1XPB). The position of each design mutation is shown with stick representation of the wild type amino acid, and is colored according to relative solvent accessibility. Yellow = surface residues (relative solvent accessibility of >= 20%). Red = core residue (relative solvent accessibility of < 20%). Positions that are not mutated are in cartoon representation in silver.

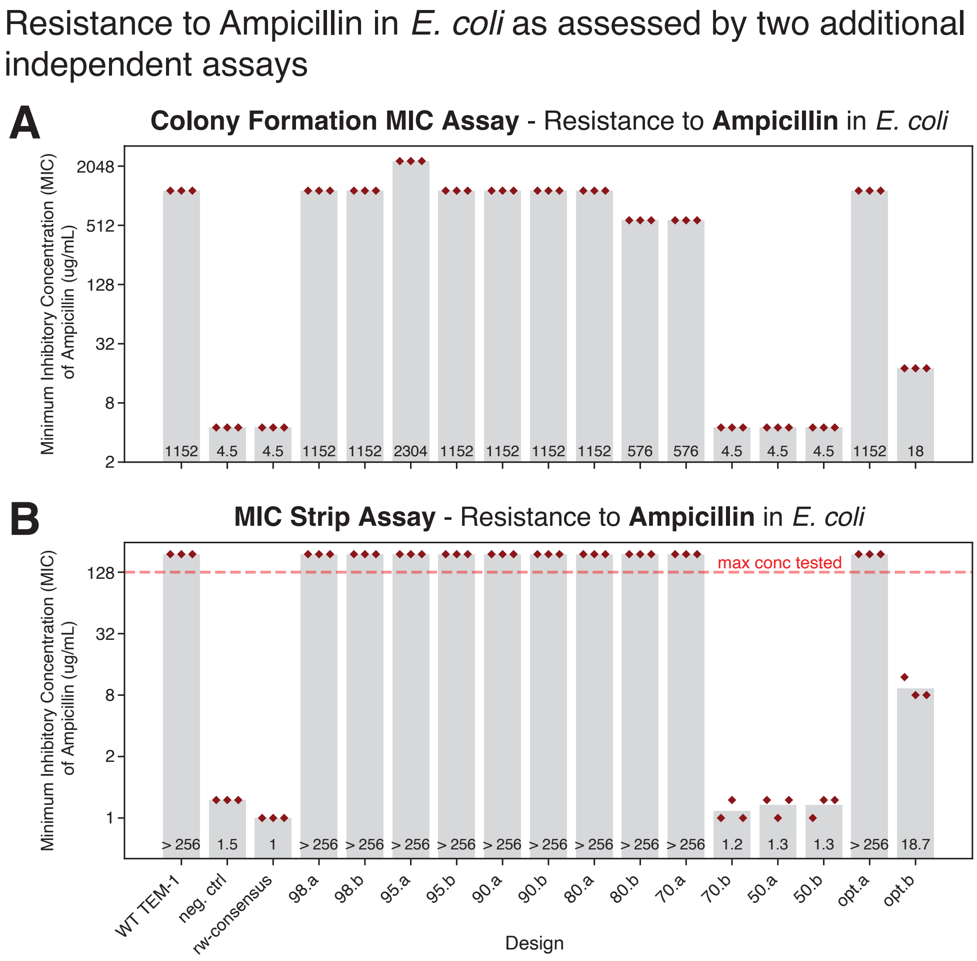

Supplementary Figure 7. Ability of designs to confer resistance to ampicillin in *E. coli*. In addition to the broth microdilution assay in the main text (Figure 3), two independent assays were used to measure minimum inhibitory concentration (MIC) of the canonical β-lactam substrate ampicillin. Resistance to ampicillin as assessed by **[A]** ability to form colonies across a serial dilution of ampicillin in MH agar, and **[B]** a MIC strip assay (Liofilchem). The maximum concentration of ampicillin tested, and therefore maximum MIC resolution, in the MIC strip assay in [B] was 256 μg/mL (indicated in red). Gray bars: mean of three replicates (red diamonds). The relative MIC between sequences was largely in agreement with the broth microdilution assay in the main text (Figure 3A). Source data are provided in the Source Data file.

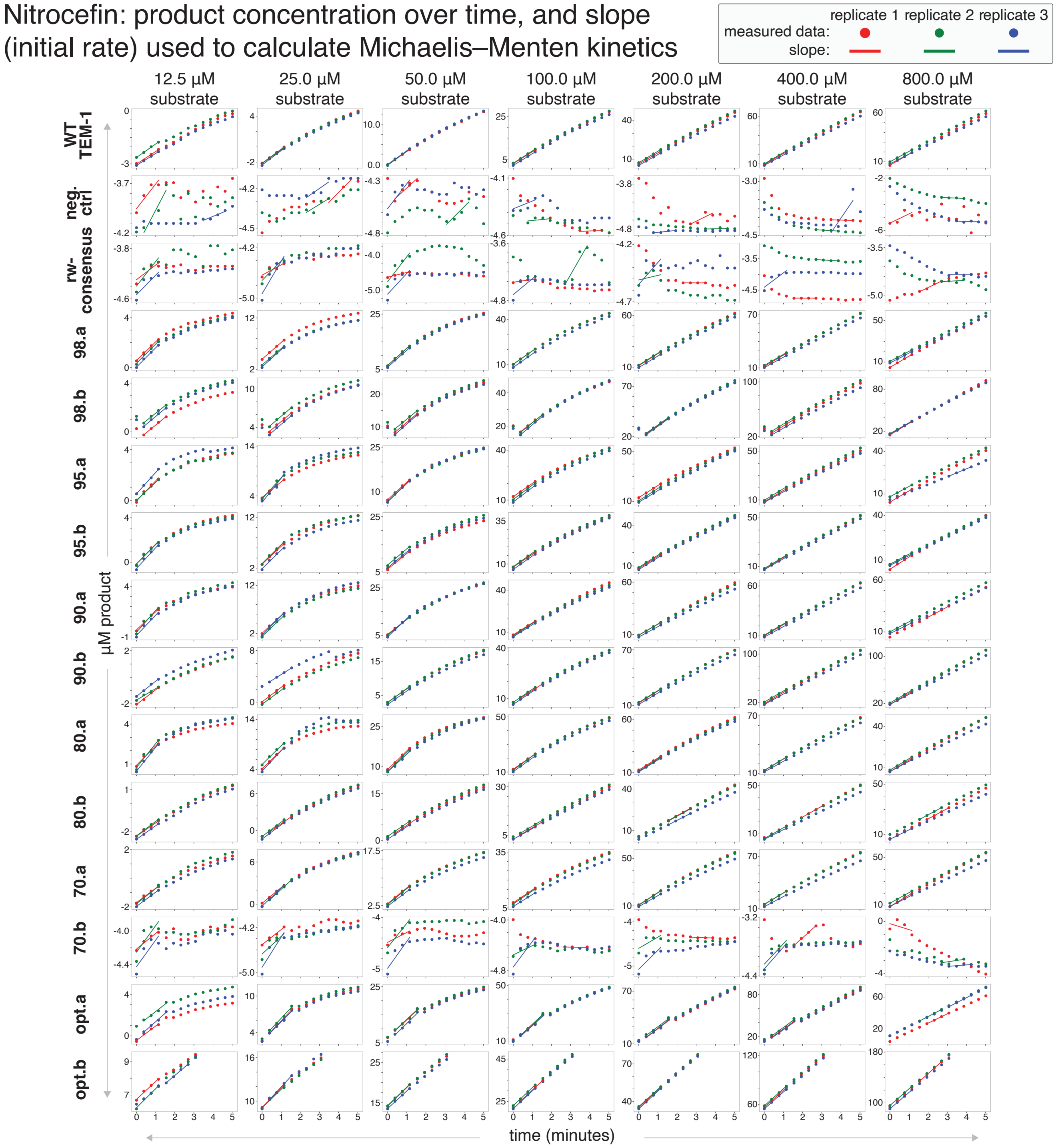

Supplementary Figure 8. Nitrocefin hydrolysis product concentration over time as a function of initial substrate concentration. Rows are each tested enzyme. Columns contain the initial substrate (nitrocefin) concentration. Dots are the absolute measured product concentration. Lines overlaid on the dots show the initial reaction rate used for fitting the Michaelis-Menton equation. Each replicate is shown in a different color (red: replicate 1, green: replicate 2, blue: replicate 3). No hydrolysis was detectable for neg. ctrl, consensus, and 70.b. Designs 50.a and 50.b were unable to be purified. Source data are provided in the Source Data file.

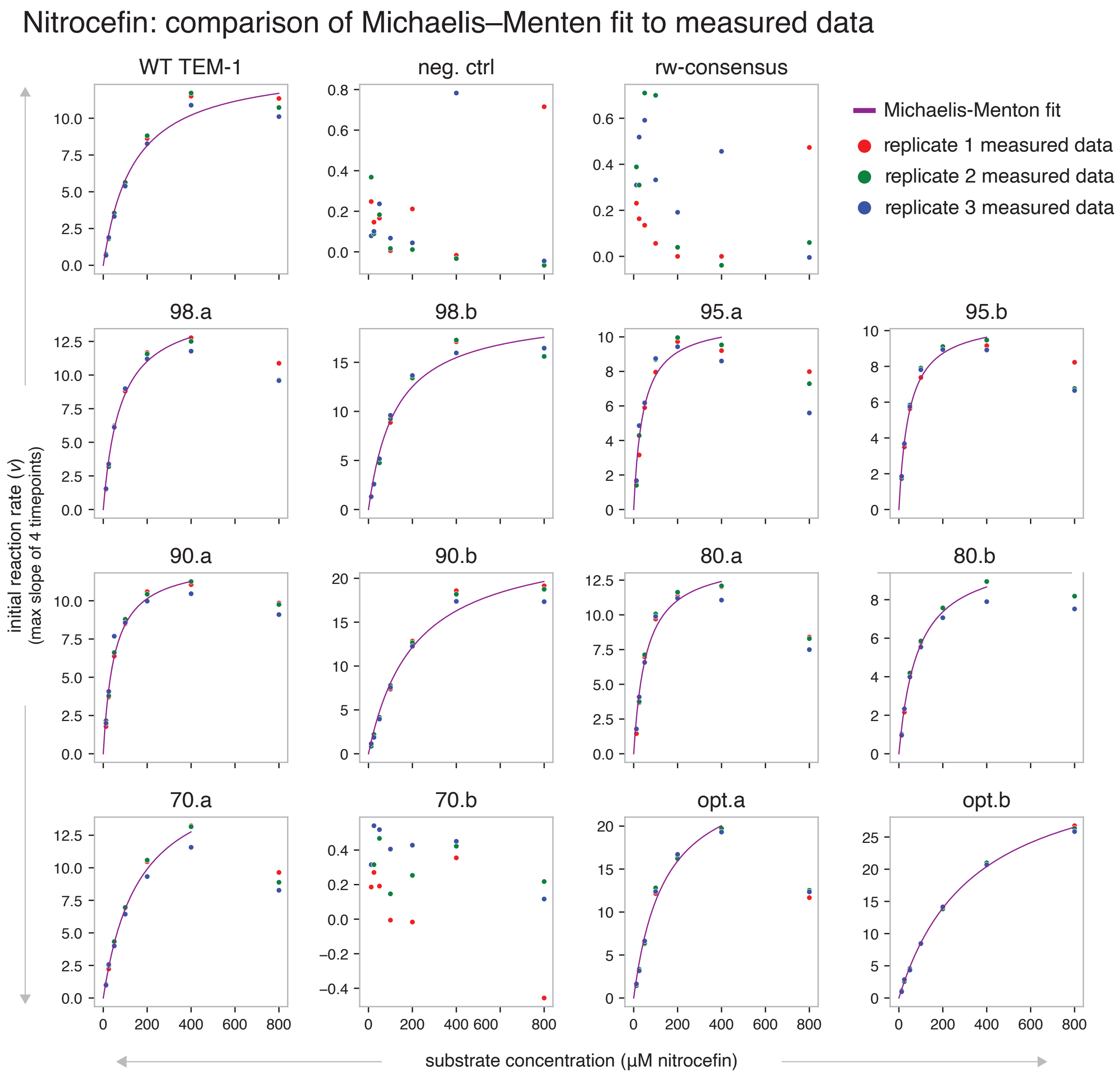

Supplementary Figure 9. Comparison of Michaelis-Menten fit to measured data. Each plot contains data from a single tested sequence. Y-axis: initial reaction rate. X-axis: substrate (nitrocefin) concentration. Dots: measured data used to fit the Michaelis-Menten equation (red: replicate 1, green: replicate 2, blue: replicate 3). Purple line: fitted results (predicted initial reaction rate as a function of nitrocefin concentration) plotted to the maximum substrate concentration used for fitting (400 μM or 800 μM, Methods). No hydrolysis was detectable for neg. ctrl, consensus, and 70.b. Designs 50.a and 50.b were unable to be purified. Source data are provided in the Source Data file.

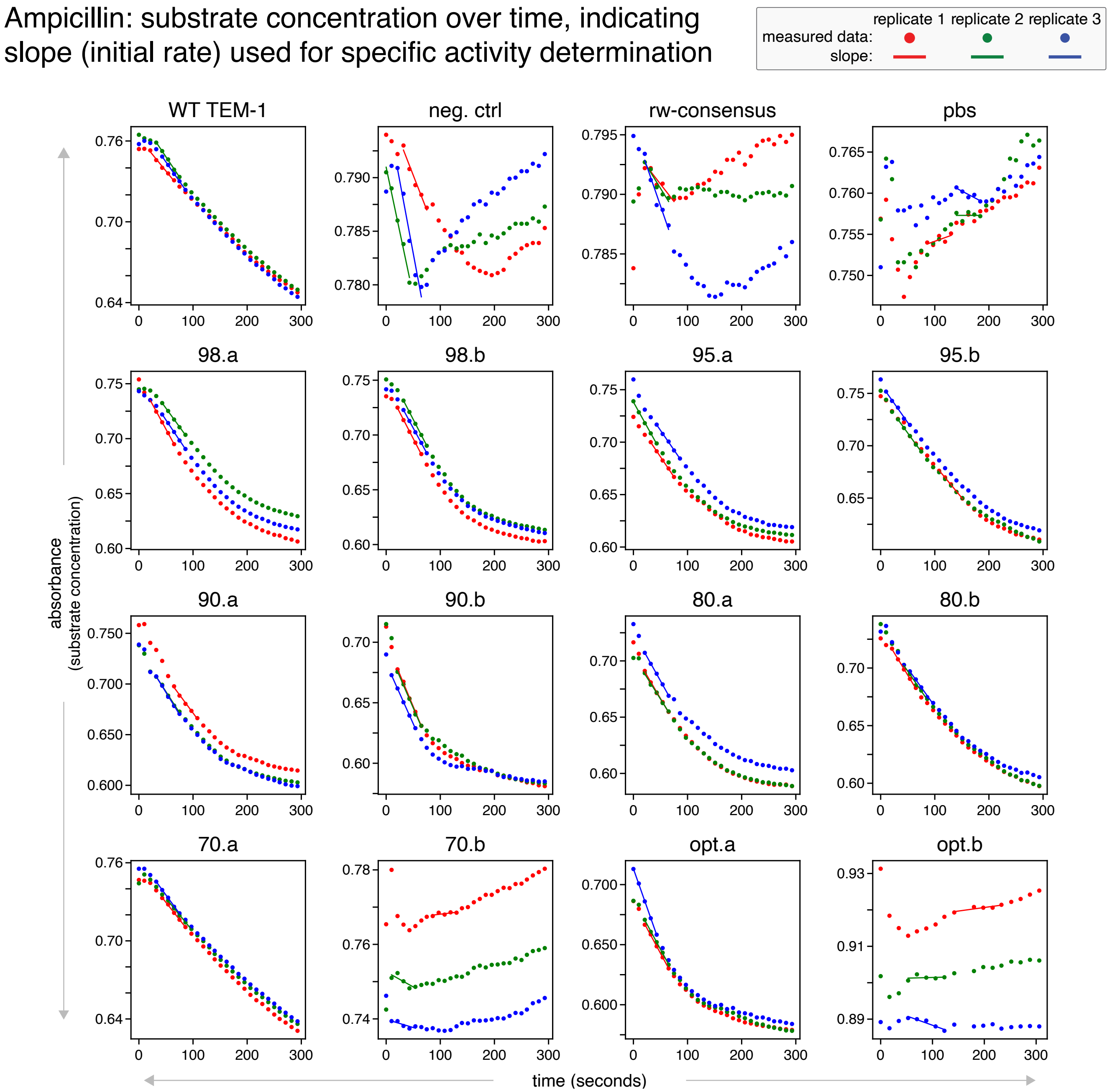

Supplementary Figure 10. Ampicillin hydrolysis substrate concentration over time. Each plot contain data from a single tested sequence. X-axis: time in seconds. Y-axis: absorbance measured at 235 nm. Dots are measured data, and the overlaid lines show the initial rate (linear regression of five timeopints) for each of the three replicates. Each replicate is shown in a different color (red: replicate 1, green: replicate 2, blue: replicate 3). No hydrolysis was detectable for neg. ctrl, consensus, and 70.b. Designs 50.a and 50.b were unable to be purified. Source data are provided in the Source Data file.

Thermostability: Differential Scanning Fluorimetry (DSF) melt curves

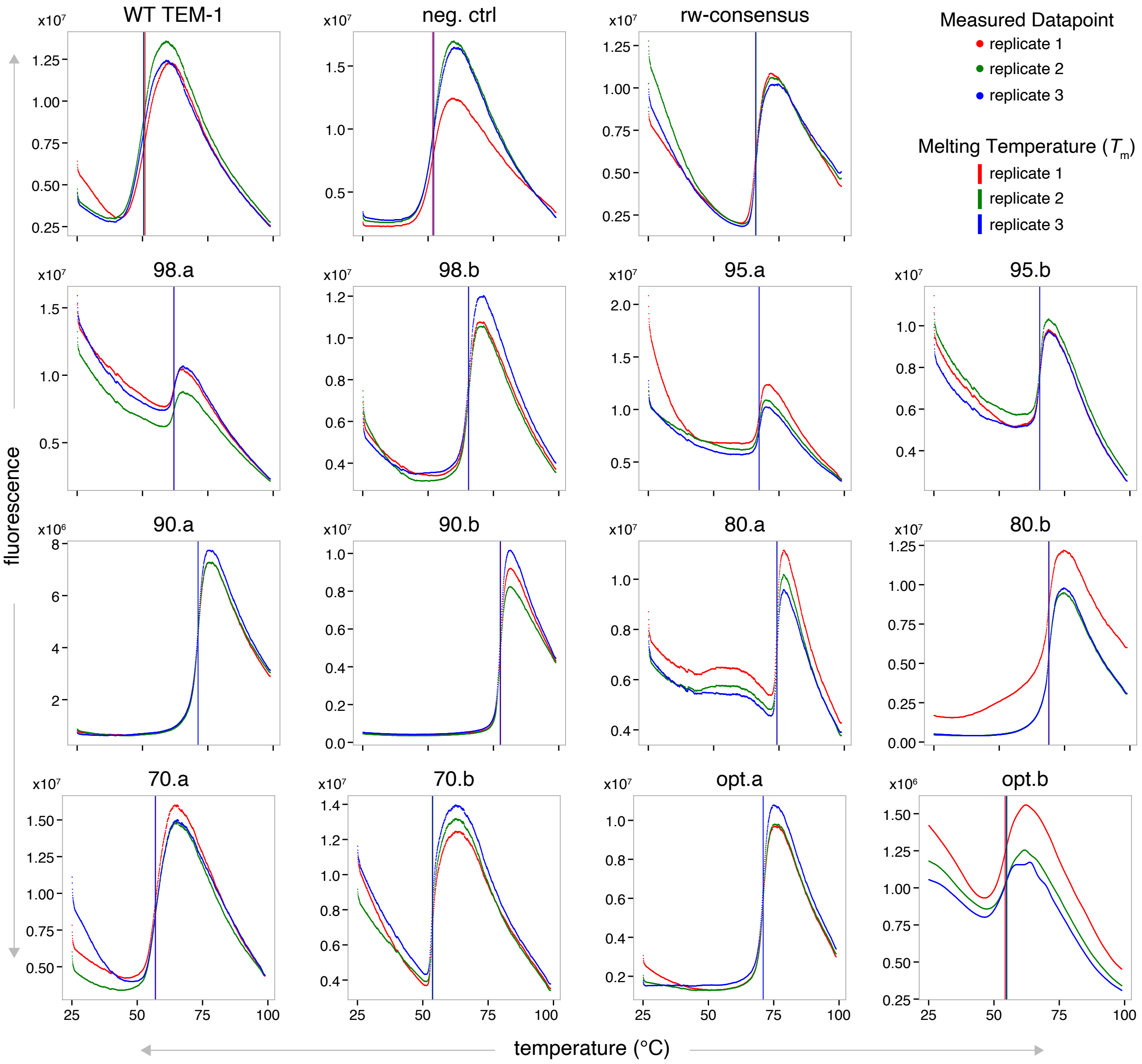

Supplementary Figure 11. Melting curves showing fluorescence emission changes with temperature in a Differential Scanning Fluorimetry assay to quantify thermal stability. Purified protein was incubated with SYPRO Orange dye to monitor protein unfolding (Methods). Melting temperature (T_m) is quantified as the midpoint of the transition curve (vertical lines). Colors depict replicate 1 (red), replicate 2 (green), and replicate 3 (blue). Source data are provided in the Source Data file.

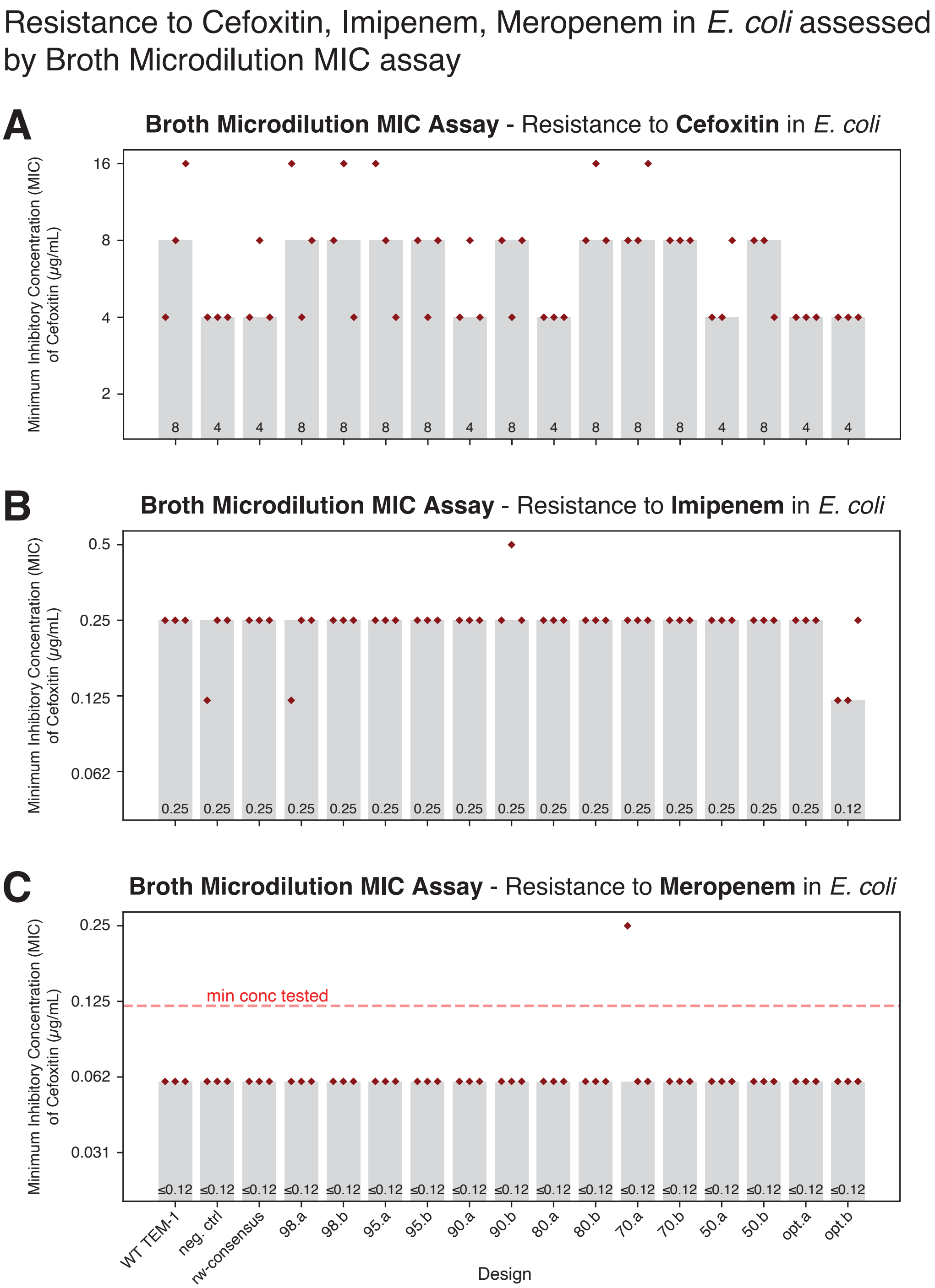

Supplementary Figure 12. For some β-lactam antibiotics, the designs showed no consistent differences in their ability to confer resistance with WT TEM-1 or the negative controls. Minimum inhibitory concentration (MIC) in *E. coli* was determined by a Clinical and Laboratory Standards Institute (CLSI) broth microdilution assay. The aggregated MIC calls (gray bars, see Methods) summarize three individual replicate experiments. **[A]** Resistance to cefoxitin, a second-generation cephamycin β-lactam antibiotic. **[B]** Resistance to imipenem and **[C]** meropenem, both of which are members of the carbapenem class of β-lactam antibiotics. The aggregated MIC calls (gray bars, see Methods) summarize three individual replicate experiments (red diamonds). Source data are provided in the Source Data file.

Resistance to Aztreonam, Ceftazidime, Cephalothin in *E. coli* assessed by MIC Strip Assay

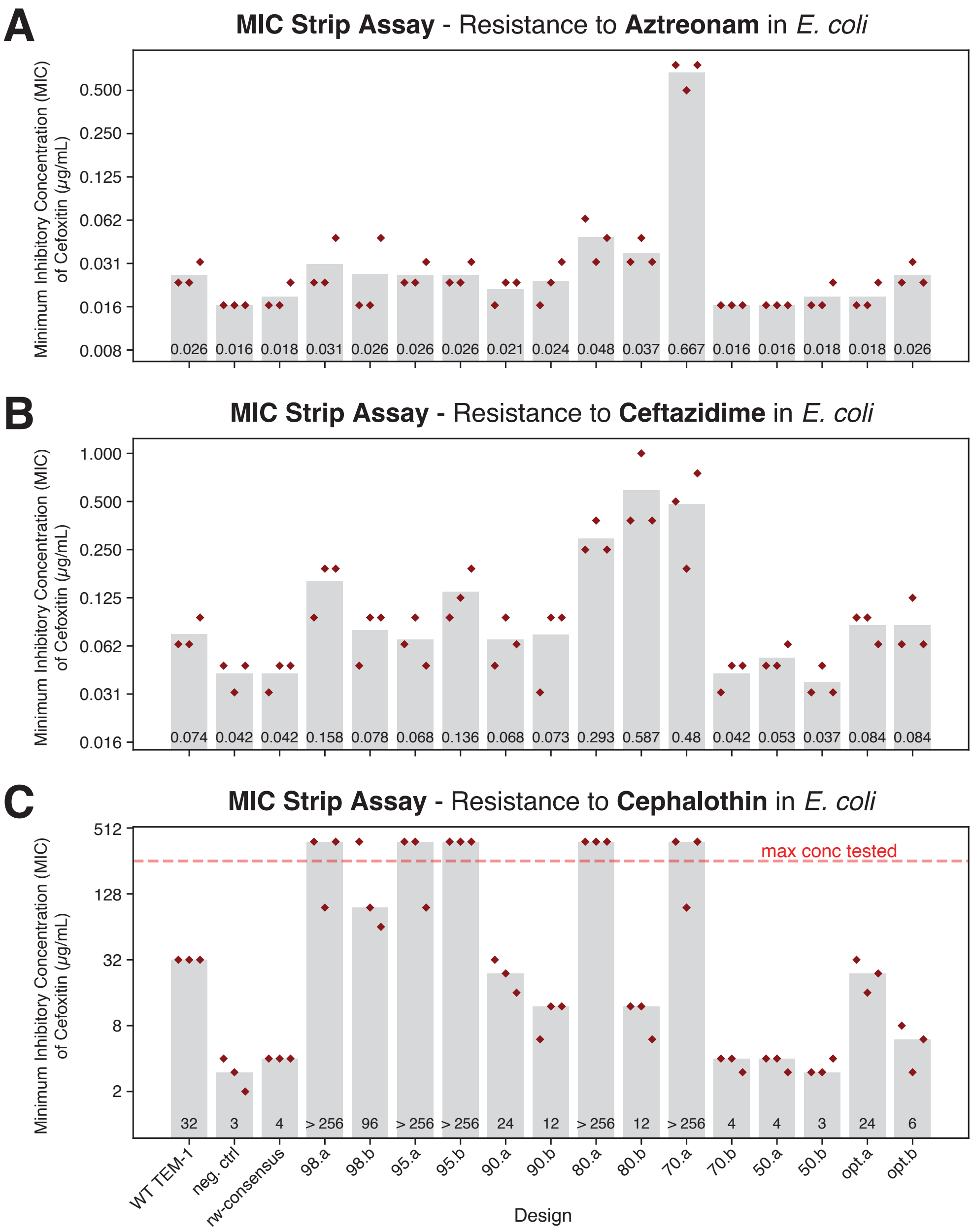

Supplementary Figure 13. Ability of designs to confer resistance to β-lactam antibiotics aztreonam, ceftazidime, and cephalothin in *E. coli*. Minimum inhibitory concentration (MIC) in E. coli was determined by a MIC strip assay (Liofilchem). **[A]** MIC of aztreonam. Gray bars: mean of three replicates (red diamonds). **[B]** MIC of ceftazidime. Gray bars: mean of three replicates (red diamonds). **[C]** MIC of cephalothin. For cephalothin, several of the replicates exceeded the maximum concentration tested (256 μg/mL), so the gray bars are either the mode of the replicates or, if there was no mode, the median of the three replicates (red diamonds). The relative MIC between sequences was largely in agreement with the broth microdilution resistance assays in the main text (Figure 4B). Source data are provided in the Source Data file.

Composite omit maps of the refined X-ray crystal structures for designs 70.a, 80.a and 80.b

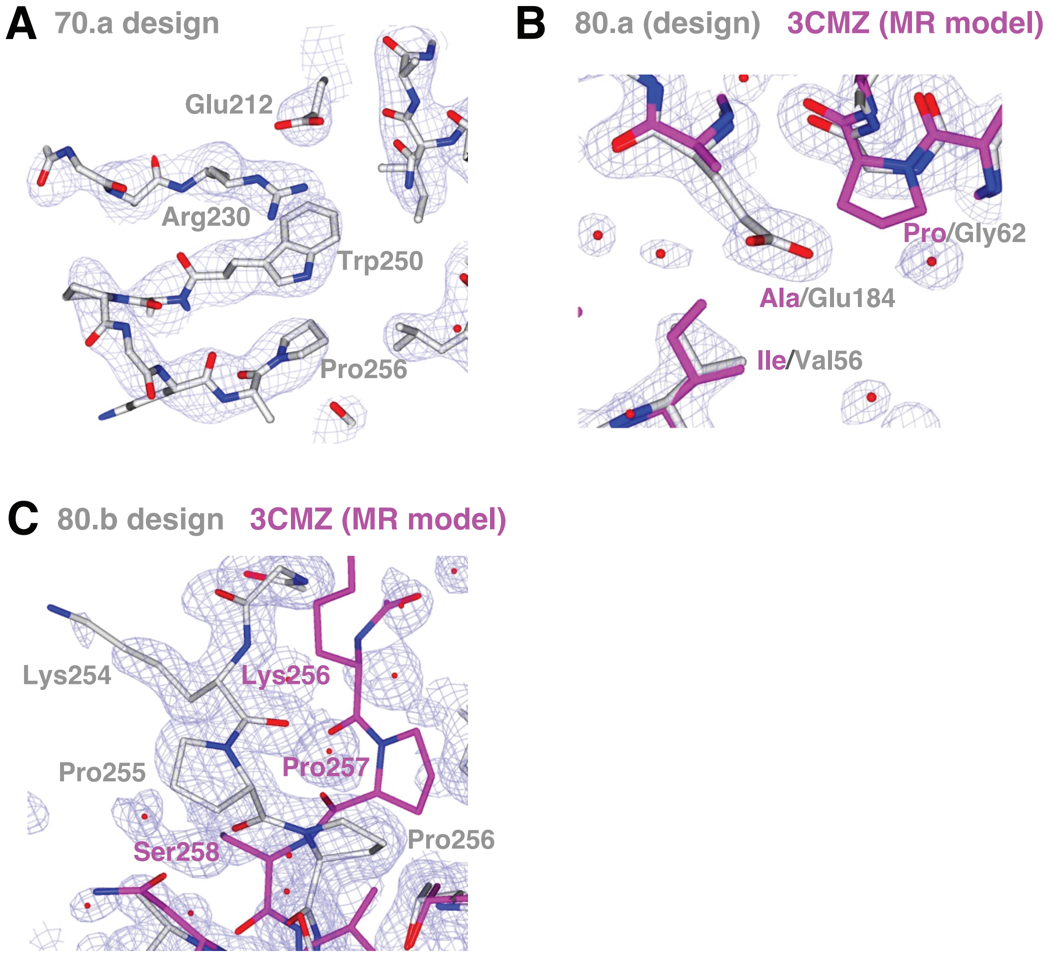

Supplementary Figure 14. Composite omit maps of refined design structures. [A] Composite omit map of the refined structure of 70.a. Cartesian simulated annealing was performed together with systematic deletions in 70.a, as implemented in Phenix. There is clear density for Trp250 and its interaction with Arg230, as discussed in the manuscript. The 2Fo-Fc map is contoured at 1σ. **[B]** Composite omit map of the refined structure of 80.a (light grey). The MR model, 3CMZ, is superposed with magenta carbon bond vectors. The 2Fo-Fc electron density corresponds to 80.a, contoured at 1σ. The sequence differences are apparent from the electron density map, suggesting that the final model of 80.5 variant is not significantly biased toward the starting 3CMZ model. **[C]** Composite omit map of the refined structure of 80.b (light grey). The full MR model 3CMZ (magenta) was superposed using secondary structure matching in Coot. The 2fo-Fc electron density map is clearly favoring the conformation of the refined 80.b structure. Pro258 (80.6) also shows clear density relative to its counterpart, Ser256 (3CMZ). The sequences for this matching motif differ due to an insertion in 80.b.

Difference Distance Matrix: comparison of residue-residue distances between 80.a and WT TEM-1

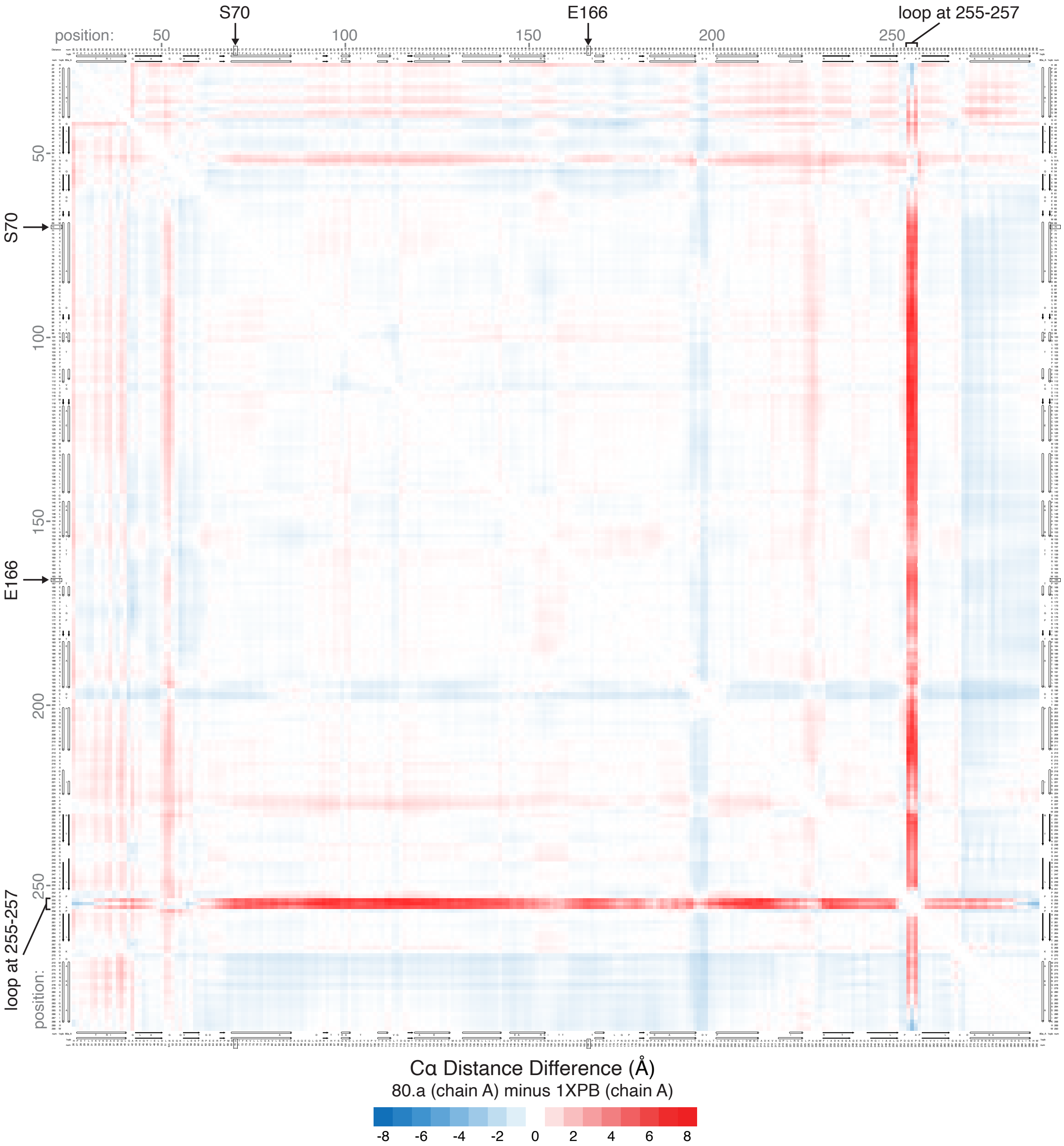

Supplementary Figure 15. Difference Distance Matrix comparing 80.a (chain A) to WT TEM-1 (PDB: 1XPB chain A). The distance between each pair of Cα atoms in both the 80.a and WT TEM-1 structures was calculated, and then the difference was computed by subtracting the WT TEM-1 distance from the 80.a distance. The only region substantially different is in a loop between positions 255-257. The Difference Distance Matrix for chain B of 80.a (compared to WT TEM-1) is nearly identical (not shown). Colors: Red indicates the distance between residue pairs is larger in 80.a than in WT TEM-1. Blue indicates the distance between residue pairs is larger in WT TEM-1 than in 80.a. Intermediate colors quantified as shown in in the colorbar. Source data are provided in the Source Data file.

Difference Distance Matrix: comparison of residue-residue distances between 80.b and WT TEM-1

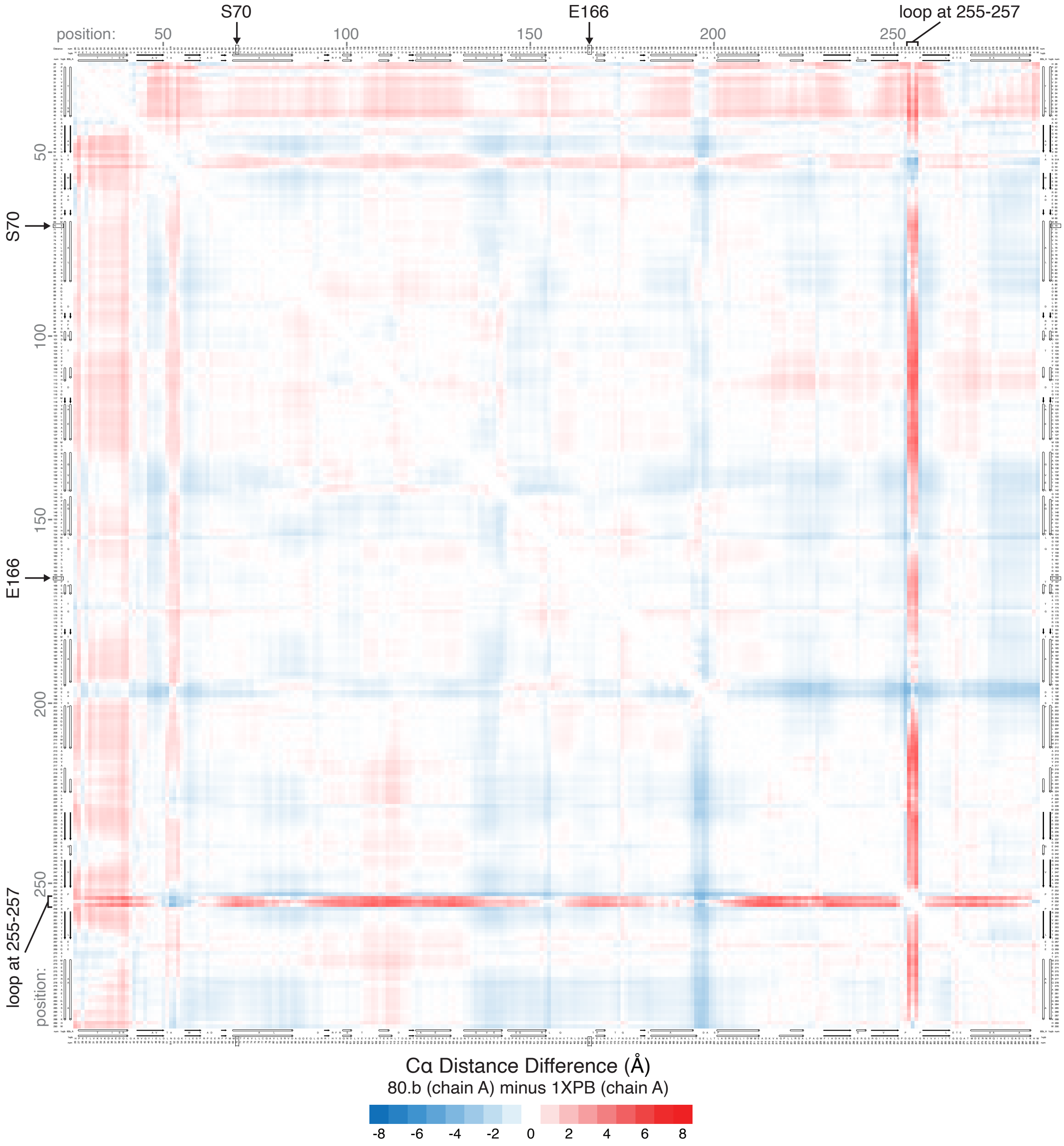

Supplementary Figure 16. Difference Distance Matrix comparing 80.b (chain A) to WT TEM-1 (PDB: 1XPB chain A). The distance between each pair of Cα atoms in both the 80.b and WT TEM-1 structures was calculated, and then the difference was computed by subtracting the WT TEM-1 distance from the 80.b distance. The only region substantially different is in a loop between positions 255-257. The Difference Distance Matrix for chain B of 80.b (compared to WT TEM-1) is nearly identical (not shown). Colors: Red indicates the distance between residue pairs is larger in 80.b than in WT TEM-1. Blue indicates the distance between residue pairs is larger in WT TEM-1 than in 80.b. Intermediate colors quantified as shown in in the colorbar. Source data are provided in the Source Data file.

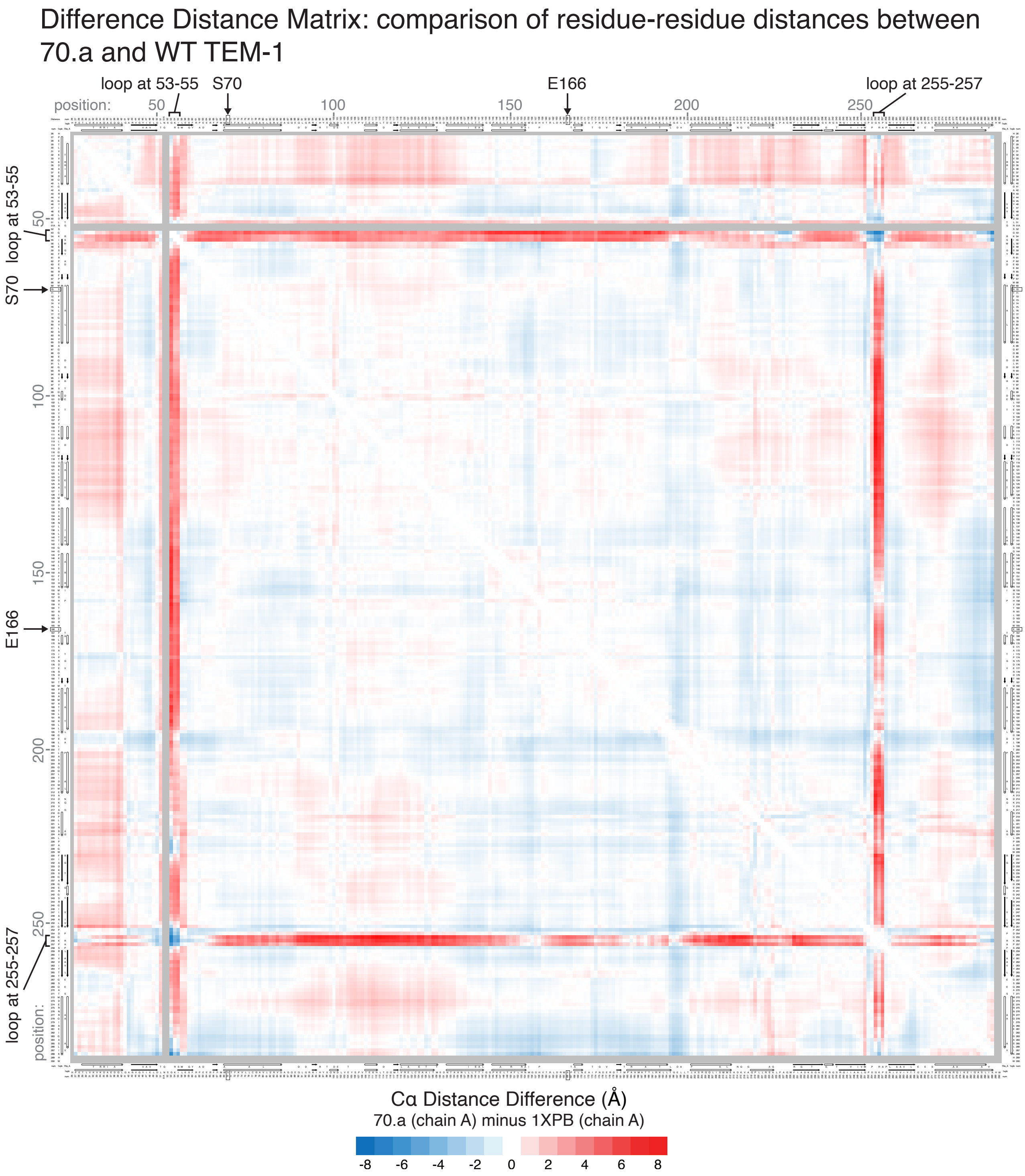

Supplementary Figure 17. Difference Distance Matrix comparing 70.a (chain A) to WT TEM-1 (PDB: 1XPB chain A). The distance between each pair of Cα atoms in both the 70.a and WT TEM-1 structures was calculated, and then the difference was computed by subtracting the WT TEM-1 distance from the 70.a distance. The only regions substantially different occur in two loops (positions 53-55 and 255-257). The Difference Distance Matrix for chain B of 70.a (compared to WT TEM-1) is nearly identical (not shown). Colors: Red indicates the distance between residue pairs is larger in 70.a than in WT TEM-1. Blue indicates the distance between residue pairs is larger in WT TEM-1 than in 70.a. Intermediate colors quantified as shown in in the colorbar. Source data are provided in the Source Data file.

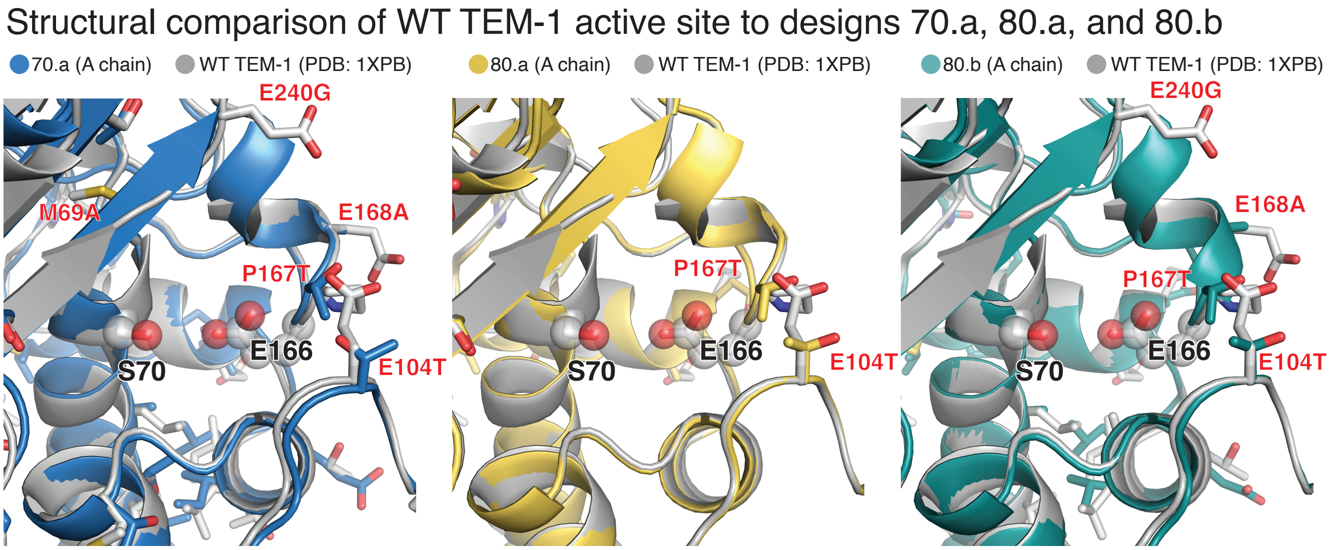

Supplementary Figure 18. Structural assessment of active site in X-ray crystal structures to WT TEM-1. Focused view of the active site of each design structure (left: 70.a, middle: 80.a, right: 80.b) aligned to WT TEM-1 (PDB: 1XPB). Catalytic residues S70 and E166 are shown in sticks with translucent spheres. Residues that are different between WT TEM-1 and the designs are highlighted as sticks and red labels indicate the mutation.

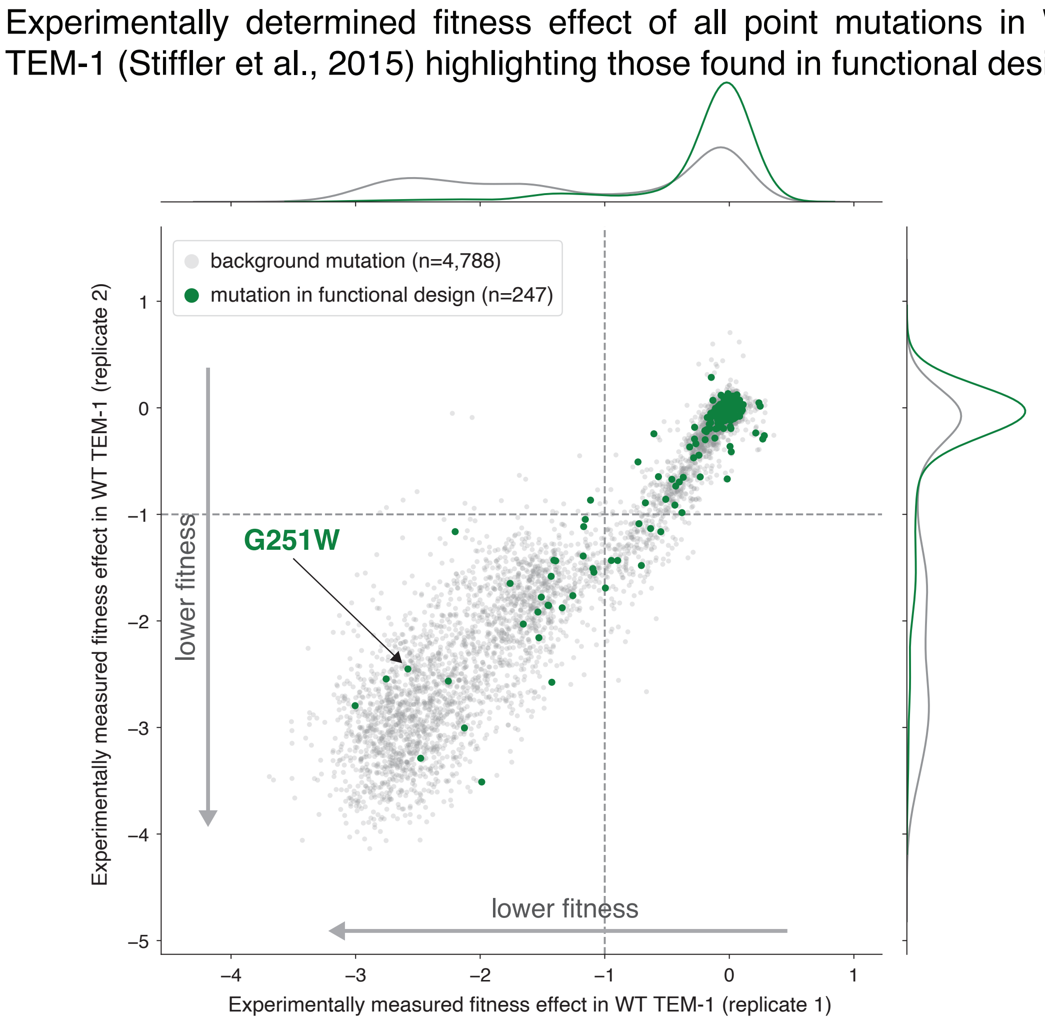

Supplementary Figure 19. Functional design variants contain mutations that negatively affect fitness in WT TEM-1 in a previously published deep mutational scan by Stiffler et al.[1]. Comparison of aggregated mutations from every functional design (70.a, 80.a, 80.b, 90.a, 90.b, 95.a, 95.b, 98.a, 98.b, opt.a, opt.b) to the experimentally-determined fitness effect all possible point mutations in the modeled WT TEM-1 sequence background (252 positions were modeled with 19 possible substitutions each). Mutations in positions that were tested in the deep mutational scan, but were not not accounted for in the model (i.e., 11 positions: 26, 27, 28, 29, 30, 31, 58, 241, 288, 289, 290), were excluded from the plot. Each dot represents a single amino acid change, and axes quantitate the fitness effect at 2,500 μg/mL ampicillin (x-axis = replicate 1, y-axis = replicate 2). Dotted lines indicate a score of less than -1, which conceptually equates to a 10-fold decrease in fitness at a given ampicillin concentration relative to WT TEM-1. Several design variants (80.a, 80.b, 70.a, and opt.a) contain mutations that negatively affect fitness in isolation in WT TEM-1, i.e., have mutations that are represented in the lower left quadrant. Marginal distributions of the data are shown using kernel density estimation (KDE) on the top (replicate 1) and the right (replicate 2) of the scatter plots. [Colors] Gray: all point mutations for positions aligned in the β-lactamase multiple sequence alignment. Green: amino acid changes that occur in at least one functional design. Source data are provided in the Source Data file.

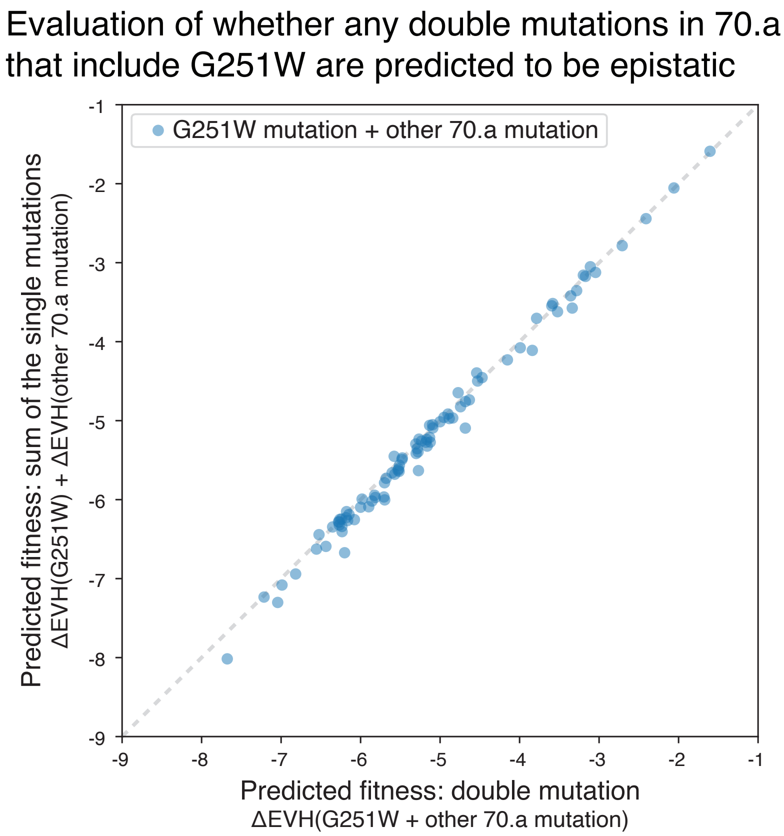

Supplementary Figure 20. Comparison of the predicted fitness of double mutations versus the sum of the predicted fitness of single mutations for G251W with other 70.a mutations on the WT TEM-1 sequence background. Each blue dot is a single 70.a mutation combined with the G251W mutation as a double mutation (x-axis) or the sum of the single mutations (y-axis). The similarity between these fitness predictions (Pearson r = 0.996) and lack of outliers suggests a general lack of epistasis between G251W and the other 70.a mutations. Source data are provided in the Source Data file.

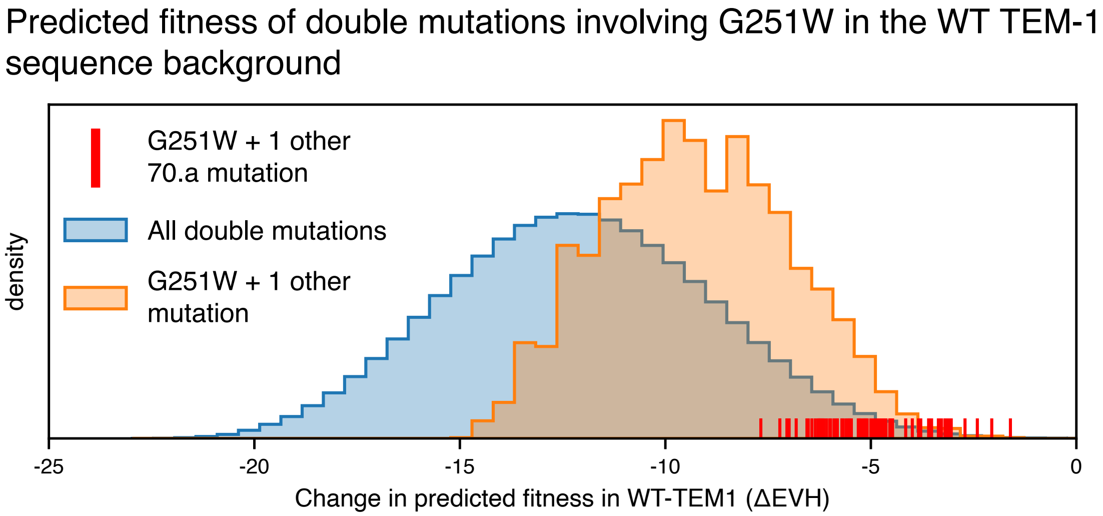

Supplementary Figure 21. Comparison of the predicted fitness changes of double mutations in the WT TEM-1 sequence background with and without the G251W mutation. The change in predicted fitness of pairs of mutations is calculated as the EVH of the double mutants minus the EVH of WT TEM-1. Blue distribution: all possible double mutations in WT TEM-1. Orange distribution: all double mutations that contain G251W. Red residual dashes show all double mutations with G251W and each 70.a mutation (n=87 doubles). The double mutations containing G251W are generally higher than random double mutations (orange versus blue), and individual mutations in 70.a when combined with G251W are on the right side of both distributions indicating they are amongst the most beneficial of double mutations. Source data for the G251W data (red dashes and orange distribution) and a random subset of all double mutations (blue distribution) are provided in the Source Data file. All double mutations can be calculated using scripts available at https://github.com/gauthierscience/beta-lac-protein-design[2].

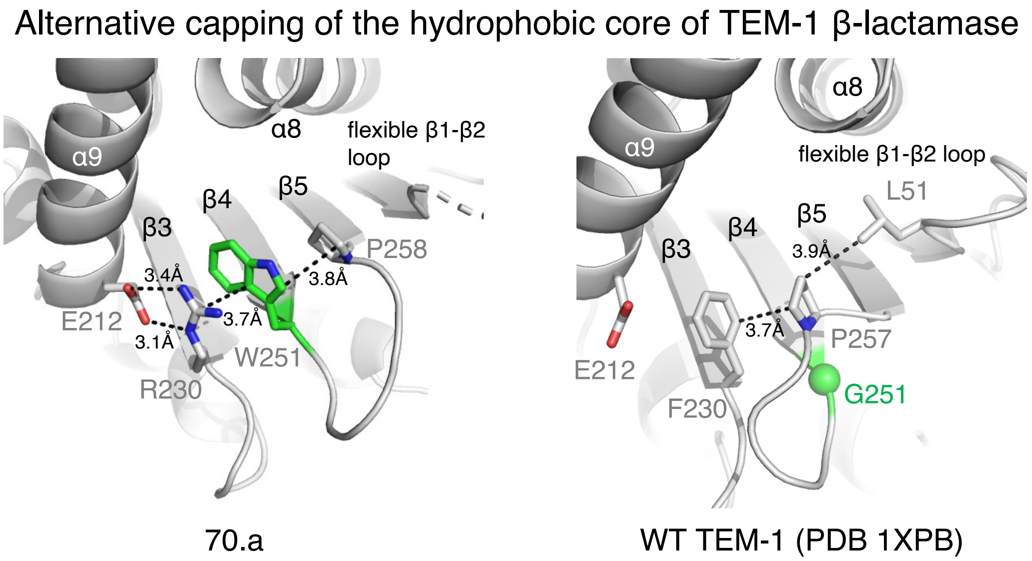

Supplementary Figure 22. Alternative capping of the hydrophobic core of TEM-1 β-lactamase. Focused visualization of position 251 in the crystal structure of the 70.a design (left) and a previously published WT TEM-1 crystal structure (right, PDB: 1XPB). The 70.a design contains the G251W mutation which, when introduced as a point mutation in WT TEM-1, is inactivating[1,3]. In the WT TEM-1 structure, G251 is localized to the tip of a β-strand. Two residues – F230 and P257 – occupy the vacant space adjacent to G251, effectively capping the hydrophobic core on this side of WT TEM-1. In the 70.a design, there are backbone and side chain conformational rearrangements that enable the indole side chain of W251 to cap the hydrophobic core. The conformation of W251 appears to be stabilized through guanidino/indole stacking interactions with R230, which in turn forms a salt bridge with E212. In summary, alternative local interactions (W251/R230/E212) may contribute to maintaining β-lactamase structure and function.

	K_m μM	k_{cat} minute^{-1}	k_{cat}/K_m μM^{-1} * minute^{-1}
WT TEM-1	136 ± 18	13689 ± 605	100 ± 14
neg. ctrl		no hydrolysis	
rw-consensus		no hydrolysis	
98.a	78 ± 7	15307 ± 462	197 ± 18
98.b	124 ± 15	20276 ± 832	164 ± 21
95.a	42 ± 7	11026 ± 521	263 ± 44
95.b	46 ± 4	10739 ± 272	233 ± 21
90.a	50 ± 5	12665 ± 377	254 ± 26
90.b	207 ± 25	24724 ± 1173	119 ± 16
80.a	56 ± 7	14153 ± 547	251 ± 32
80.b	85 ± 7	10501 ± 308	124 ± 11
70.a	163 ± 15	17951 ± 758	110 ± 11
70.b		no hydrolysis	
50.a		unable to purify	
50.b		unable to purify	
opt.a	139 ± 15	27040 ± 1215	194 ± 22
opt.b	342 ± 11	37894 ± 539	111 ± 4

Supplementary Table 1. Michaelis-Menton parameters of Nitrocefin hydrolysis. All values rounded to nearest integer. Number following $\pm$ indicates standard error, which was derived directly from the model fit (Python *lmfit* module) for k_{cat} and K_m, or calculated using error propagation for k_{cat}/K_m (Methods).

	**70.a**	**80.a**	**80.b**
MR search model	1XPB	3CMZ	3CMZ
Crystallization	0.02M Divalent II, 0.1M buffer system 6, pH 8.5, 30% precipitant mix 7 (Morpheus II screen B9)	30% PEG3350 0.1M MES pH 6.5	30% PEG3350 0.1M MES pH 6.5
Beamline	NECAT 24ID-C	NSLS2 FMX	NSLS2 FMX
Wavelength (Å)	0.97918	0.97932	0.97932
Space group	$P\ 4_1$	P1	$P\ 2_1\ 2_1\ 2_1$
Cell a, b, c, (Å)	114.62, 114.62, 48.88	50.92, 51.12, 56.89	59.95, 60.9, 121.88
α, β, γ (°)	90, 90, 90	78.66, 84.9, 64.87	90, 90, 90
Unique reflections	14355 (1404)	40574 (3405)	58092 (5320)
Completeness (%)	99.9 (100)	89.95 (75.38)	95.85 (88.77)
$\langle I/\sigma \rangle$	9.0 (1.1)	7.30 (1.40)	17.90 (3.40)
Multiplicity	10.2 (5.0)	2.1 (2.1)	13.6 (12.9)
R-merge	0.249 (1.832)	0.085 (0.442)	0.100 (0.723)
$CC_{1/2}$	0.947 (0.347)	0.994 (0.558)	0.999 (.887)
Refinement			
Resolution (Å)	41.9-2.90 (3.0-2.90)	27.89-1.83 (1.87-1.83)	29.57-1.59 (1.63-1.59)
Protein residues/waters	512	524	525
No. reflections for R-free	724 (69)	1895 (162)	1932 (184)
R-work	0.220 (0.3551)	0.1741 (0.2580)	0.1633 (0.1831)
R-free	0.266 (0.4188)	0.2097 (0.3195)	0.1923 (0.2263)
RMSD bond lengths (Å)	0.009	0.009	0.007
RMSD bond angles (°)	1.21	1.36	1.25
Average overall *B*-factor	62.80	20.73	16.78
Mean *B*-factors ($Å^2$) protein/ligands/solvent	63.02/61.31/50.57	19.5/29.28	15.00/27.74
Ramachandran analysis favored/allowed (%)	96.22/2.79	98.46/1.54	98.27/1.73
PDB accession code	8RQU	8GII	8GIJ

Supplementary Table 2. β-Lactamase crystallographic data and refinement statistics. Values in parentheses correspond to the statistics in the highest resolution bin. RMSD, root-mean-square deviation.

$$R_{merge} = \frac{\sum hkl \sum_{i=1}^{n} |I_i(hkl) - \bar{I}(hkl)|}{\sum hkl \sum_{i=1}^{n} I_i(hkl)}$$

References

1. Stiffler, M. A., Hekstra, D. R. & Ranganathan, R. Evolvability as a function of purifying selection in TEM-1 β-lactamase. *Cell* **160**, 882–892 (2015). URL http://dx.doi.org/10.1016/j.cell.2015.01.035. DOI 10.1016/j.cell.2015.01.035.

2. Fram, B. *et al.* Simultaneous enhancement of multiple functional properties using evolution-informed protein design (2024). URL http://dx.doi.org/10.5281/zenodo.11123122. DOI 10.5281/zenodo.11123122.

3. Birgy, A. *et al.* Local and global protein interactions contribute to residue entrenchment in Beta-Lactamase TEM-1. *Antibiot. (Basel, Switzerland)* **11** (2022). URL http://dx.doi.org/10.3390/antibiotics11050652. DOI 10.3390/antibiotics11050652.
