# Supplementary material for: Simultaneous enhancement of multiple functional properties using evolution-informed protein design
Source: Nat Commun. 2024 Jun 20;15:5141. doi: 10.1038/s41467-024-49119-x (PMC11190266; doi:10.1038/s41467-024-49119-x)
Supplement: Supplementary file 3 — Reporting Summary [file 41467_2024_49119_MOESM3_ESM.pdf]

nature portfolio

Corresponding author(s): Benjamin Fram and Nicholas Gauthier

Last updated by author(s): May 23, 2024

Reporting Summary

Nature Portfolio wishes to improve the reproducibility of the work that we publish. This form provides structure for consistency and transparency in reporting. For further information on Nature Portfolio policies, see our Editorial Policies and the Editorial Policy Checklist.

Statistics

For all statistical analyses, confirm that the following items are present in the figure legend, table legend, main text, or Methods section.

n/a	Confirmed	
☐	☒	The exact sample size (*n*) for each experimental group/condition, given as a discrete number and unit of measurement
☐	☒	A statement on whether measurements were taken from distinct samples or whether the same sample was measured repeatedly
☒	☐	The statistical test(s) used AND whether they are one- or two-sided<br>*Only common tests should be described solely by name; describe more complex techniques in the Methods section.*
☒	☐	A description of all covariates tested
☒	☐	A description of any assumptions or corrections, such as tests of normality and adjustment for multiple comparisons
☐	☒	A full description of the statistical parameters including central tendency (e.g. means) or other basic estimates (e.g. regression coefficient) AND variation (e.g. standard deviation) or associated estimates of uncertainty (e.g. confidence intervals)
☒	☐	For null hypothesis testing, the test statistic (e.g. *F*, *t*, *r*) with confidence intervals, effect sizes, degrees of freedom and *P* value noted<br>*Give P values as exact values whenever suitable.*
☒	☐	For Bayesian analysis, information on the choice of priors and Markov chain Monte Carlo settings
☒	☐	For hierarchical and complex designs, identification of the appropriate level for tests and full reporting of outcomes
☐	☒	Estimates of effect sizes (e.g. Cohen's *d*, Pearson's *r*), indicating how they were calculated

Our web collection on statistics for biologists contains articles on many of the points above.

Software and code

Policy information about availability of computer code

Data collection	The following software was used for data collection: Applied Biosystems Protein Thermal Shift software version 1.2, blastp 2.14.1, jackhmmer - 3.2.1, MATLAB 9.4 R2018a.
Data analysis	The following was used for data analysis: EVcouplings 0.1.2 (currently development version on github), Python 3.9.17, PyMOL 2.5.2, XDS February 5, 2021, Aimless 0.5.32, PHENIX 1.20.1, COOT 9.8.6, pandas 2.0.3, biopython 1.81, seaborn 0.12.2, openpyxl 3.1.2, scikit-learn 1.3.0, jupyter 1.0.0, lmfit 1.2.2, numpy 1.23.5. Custom data analysis software can be found at https://github.com/gauthierscience/beta-lac-protein-design [DOI: 10.5281/zenodo.11123122].

For manuscripts utilizing custom algorithms or software that are central to the research but not yet described in published literature, software must be made available to editors and reviewers. We strongly encourage code deposition in a community repository (e.g. GitHub). See the Nature Portfolio guidelines for submitting code & software for further information.

Data

Policy information about availability of data

All manuscripts must include a data availability statement. This statement should provide the following information, where applicable:

- Accession codes, unique identifiers, or web links for publicly available datasets
- A description of any restrictions on data availability
- For clinical datasets or third party data, please ensure that the statement adheres to our policy

There are no restrictions on data access. All source data used for figures in this study are provided in the Source Data file provided by the journal. All code, raw and

processed data are available at https://github.com/gauthierscience/beta-lac-protein-design [DOI: 10.5281/zenodo.11123122].

Accession codes generated by this study:

Crystal structures
The crystal structures generated in this study have been deposited in the RCSB Protein Data Bank (RCSB PDB) under accession codes 8RQU [https://doi.org/10.2210/pdb8RQU/pdb] (70.a), 8GII [https://doi.org/10.2210/pdb8GII/pdb] (80.a), and 8GIJ [https://doi.org/10.2210/pdb8GIJ/pdb] (80.b).

Plasmids
The plasmids generated in this study have been deposited at Addgene under accession codes 202332 [http://n2t.net/addgene:202332] (98.a), 202333 [http://n2t.net/addgene:202333] (98.b), 202334 [http://n2t.net/addgene:202334] (95.a), 202335 [http://n2t.net/addgene:202335] (95.b), 202336 [http://n2t.net/addgene:202336] (90.a), 202337 [http://n2t.net/addgene:202337] (90.b), 202338 [http://n2t.net/addgene:202338] (80.a), 202339 [http://n2t.net/addgene:202339] (80.b), 202340 [http://n2t.net/addgene:202340] (70.a), 202341 [http://n2t.net/addgene:202341] (70.b), 202342 [http://n2t.net/addgene:202342] (50.a), 202343 [http://n2t.net/addgene:202343] (50.b), 202347 [http://n2t.net/addgene:202347] (opt.a), 202348 [http://n2t.net/addgene:202348] (opt.b), 202349 [http://n2t.net/addgene:202349] (rw-consensus), 202350 [http://n2t.net/addgene:202350] (neg. ctrl), 202351 [http://n2t.net/addgene:202351] (WT TEM-1).

Nucleotide sequences
The nucleotide sequences used in this study are available in the Source Data and have been deposited at GenBank under accession codes PP763450 [https://www.ncbi.nlm.nih.gov/nucleotide/PP763450] (WT TEM-1), PP763457 [https://www.ncbi.nlm.nih.gov/nucleotide/PP763457] (neg. ctrl), PP763449 [https://www.ncbi.nlm.nih.gov/nucleotide/PP763449] (rw-consensus), PP763456 [https://www.ncbi.nlm.nih.gov/nucleotide/PP763456] (98.a), PP763452 [https://www.ncbi.nlm.nih.gov/nucleotide/PP763452] (98.b), PP763460 [https://www.ncbi.nlm.nih.gov/nucleotide/PP763460] (95.a), PP763447 [https://www.ncbi.nlm.nih.gov/nucleotide/PP763447] (95.b), PP763455 [https://www.ncbi.nlm.nih.gov/nucleotide/PP763455] (90.a), PP763453 [https://www.ncbi.nlm.nih.gov/nucleotide/PP763453] (90.b), PP763461 [https://www.ncbi.nlm.nih.gov/nucleotide/PP763461] (80.a), PP763448 [https://www.ncbi.nlm.nih.gov/nucleotide/PP763448] (80.b), PP763458 [https://www.ncbi.nlm.nih.gov/nucleotide/PP763458] (70.a), PP763446 [https://www.ncbi.nlm.nih.gov/nucleotide/PP763446] (70.b), PP763445 [https://www.ncbi.nlm.nih.gov/nucleotide/PP763445] (50.a), PP763459 [https://www.ncbi.nlm.nih.gov/nucleotide/PP763459] (50.b), PP763451 [https://www.ncbi.nlm.nih.gov/nucleotide/PP763451] (opt.a), PP763454 [https://www.ncbi.nlm.nih.gov/nucleotide/PP763454] (opt.b).

Additional accession codes used by this study:
The WT TEM-1 crystal structure is available at RCSB Protein Data Bank (RCSB PDB) under accession code 1XPB [https://doi.org/10.2210/pdb1XPB/pdb]. Accession codes for the natural multiple sequence alignment used for model generation as well as accession codes for the 542 PDB structures used in Figure 6 are available in the Source Data.

Research involving human participants, their data, or biological material

Policy information about studies with human participants or human data. See also policy information about sex, gender (identity/presentation), and sexual orientation and race, ethnicity and racism.

Reporting on sex and gender	N/A
Reporting on race, ethnicity, or other socially relevant groupings	N/A
Population characteristics	N/A
Recruitment	N/A
Ethics oversight	N/A

Note that full information on the approval of the study protocol must also be provided in the manuscript.

Field-specific reporting

Please select the one below that is the best fit for your research. If you are not sure, read the appropriate sections before making your selection.

[x] Life sciences   [ ] Behavioural & social sciences   [ ] Ecological, evolutionary & environmental sciences

For a reference copy of the document with all sections, see nature.com/documents/nr-reporting-summary-flat.pdf

Life sciences study design

All studies must disclose on these points even when the disclosure is negative.

Sample size	We chose to sample size based on experimental tractability and resource constraints, characterizing 17 variants and associated controls.
Data exclusions	No data were excluded - all tested variants are reported.

Replication	Two variants were tested at each distance constraint. All data were performed in triplicate. When possible multiple independent assays were performed to verify the same conclusion (e.g., minimum inhibitory concentration using MIC strips, broth dilutions, and colony counting). All data were found to be reproducible.
Randomization	For the selection of designs for experimental testing, two of the six sequences were selected randomly at each sequence identity cutoff. No randomization was performed during characterization as samples were not allocated into groups because each sample represented a distinct group itself.
Blinding	The assays performed in this manuscript yielded objective measurements such as thermostability, crystallography, and antibiotic resistance (MIC). The outcomes of these assays are not subject to subjective interpretation. Thus, blinding was not necessary.

Reporting for specific materials, systems and methods

We require information from authors about some types of materials, experimental systems and methods used in many studies. Here, indicate whether each material, system or method listed is relevant to your study. If you are not sure if a list item applies to your research, read the appropriate section before selecting a response.

Materials & experimental systems

n/a	Involved in the study	
☒	☐	Antibodies
☒	☐	Eukaryotic cell lines
☒	☐	Palaeontology and archaeology
☐	☒	Animals and other organisms
☒	☐	Clinical data
☒	☐	Dual use research of concern
☒	☐	Plants

Methods

n/a	Involved in the study	
☒	☐	ChIP-seq
☒	☐	Flow cytometry
☒	☐	MRI-based neuroimaging

Animals and other research organisms

Policy information about studies involving animals; ARRIVE guidelines recommended for reporting animal research, and Sex and Gender in Research

Laboratory animals	The study did not involve laboratory animals.
Wild animals	The study did not involve wild animals.
Reporting on sex	The study did not involve any animals.
Field-collected samples	The study did not involve any field collected samples.
Ethics oversight	No ethical guidance or approval was required as all experiments solely involved in vitro assays and assays in lab bacteria.

Note that full information on the approval of the study protocol must also be provided in the manuscript.

Plants

Seed stocks	The study did not involve plants.
Novel plant genotypes	The study did not involve plants.
Authentication	The study did not involve plants.
